# Spatial features for *Escherichia coli* genome organization

**DOI:** 10.1186/s12864-015-1258-1

**Published:** 2015-02-05

**Authors:** Ting Xie, Liang-Yu Fu, Qing-Yong Yang, Heng Xiong, Hongrui Xu, Bin-Guang Ma, Hong-Yu Zhang

**Affiliations:** National Key Laboratory of Crop Genetic Improvement, Agricultural Bioinformatics Key Laboratory of Hubei Province, College of Informatics, Huazhong Agricultural University, Wuhan, 430070 P. R. China

**Keywords:** *Escherichia coli*, Chromosome architecture, Genome organization, Co-expression, Protein–protein interactions

## Abstract

**Background:**

In bacterial genomes, the compactly encoded genes and operons are well organized, with genes in the same biological pathway or operons in the same regulon close to each other on the genome sequence. In addition, the linearly close genes have a higher probability of co-expression and their protein products tend to form protein–protein interactions. However, the organization features of bacterial genomes in a three-dimensional space remain elusive. The DNA interaction data of *Escherichia coli*, measured by the genome conformation capture (GCC) technique, have recently become available, which allowed us to investigate the spatial features of bacterial genome organization.

**Results:**

By renormalizing the GCC data, we compared the interaction frequency of operon pairs in the same regulon with that of random operon pairs. The results showed that arrangements of operons in the *E. coli* genome tend to minimize the spatial distance between operons in the same regulon. A similar global organization feature exists for genes in biological pathways of *E. coli.* In addition, the genes close to each other spatially (even if they are far from each other on the genome sequence) tend to be co-expressed and form protein–protein interactions. These results provided new insights into the organization principles of bacterial genomes and support the notion of transcription factory.

**Conclusions:**

This study revealed the organization features of *Escherichia coli* genomic functional units in the 3D space and furthered our understanding of the link between the three-dimensional structure of chromosomes and biological function.

**Electronic supplementary material:**

The online version of this article (doi:10.1186/s12864-015-1258-1) contains supplementary material, which is available to authorized users.

## Background

Thousands of genes are compactly encoded in bacterial genomes and orchestrate life activities, such as DNA duplication, RNA transcription and protein translation. The genes need to be well organized in the genome for effective regulation of different biological processes. Bacterial genes are not randomly distributed on the genomic sequence, but organized in sequential functional units called operons [1]. The genes in an operon tend to be co-expressed [1,2] and their protein products have higher probability to interact with each other [3,4]. Operons participating in the same biological pathway or regulon (a group of transcriptionally co-regulated operons) are also close to each other on the genome sequence and present in one or multiple clusters [5,6]. However, numerous large regulons exist comprising multiple clustered operons that are separated distantly on the genome sequence. The organization of these long-range regulons has been suggested to be related with the three-dimensional packing of the chromosome, but this remains to be examined [6].

In the past decade, the chromosome conformation capture (3C) technique and its derivatives, such as 4C, 5C, Hi-C, and TCC [7], have been developed to detect DNA–DNA interactions to infer the chromosome spatial organization. The application of this technique in eukaryotes resulted in the interpretation of contact patterns between regulatory elements in the 3D space [8,9] and provided substantial information about the principles of chromosomal organization [10,11]. However, the application of 3C techniques in prokaryotes is still in its infancy [12]. Recently, Cagliero and co-workers determined the chromosome conformation for *Escherichia coli* growing at the exponential (L) and starvation (S) phases using the genome conformation capture (GCC) technique [13]. In this study, we attempted to use these valuable datasets to investigate the spatial features of bacterial genome organization.

## Results and discussion

### Renormalization and profile of the GCC data

We renormalized the GCC datasets using the following steps. First, high-quality reads were mapped onto the reference genome (*E. coli* K12 MG1655) using bowtie2 (version 2.1.0) [14]. The resulting contact counts were further refined by setting the contact distance threshold between the contact fragments to remove self-ligation, non-ligation and random breaks (Additional file 1: Table S1). The noise was removed by setting a minimum required contact number through controlling the false discovery rate (FDR; Additional file 1: Table S2, see Additional file 2). We divided the genome into 10-kilobase (kb) bins to derive the DNA interaction information [12]. At 10 kb resolution, 84.05% of the operons and 90.86% of the genes were inside (not across) the bins. Considering that the uneven distribution of the restriction enzyme sites (RESs) can bias the interaction frequencies, we normalized the interaction frequencies by dividing the number of Hhal RESs for each bin to remove this bias (Additional file 1: Figure S1) [12].

**Figure 1:**
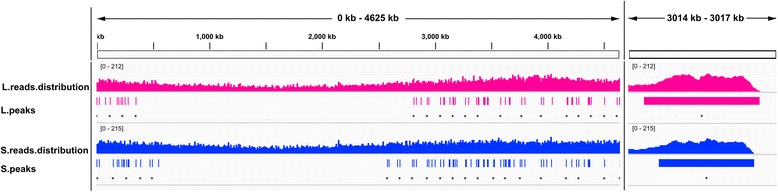
**The IGV display of reads mapping for exponential (L) phase and starvation (S) phases.** The “distribution” tracks show the density of mapped reads along the genome, visualized by Integrative Genome Viewer Integration (IGV). The “peaks” tracks show a high mapping frequency of reads to a particular region (examples indicated by asterisks). Red and blue represent the L phase and S phase, respectively. As shown to the right of this figure, the region of the selected PEAK (No. 45 in L, No. 49 in S) is shown as an example (3014–3017 kb). The middle section of the peak illustrates the pileup signal in the top track.

**Figure 2:**
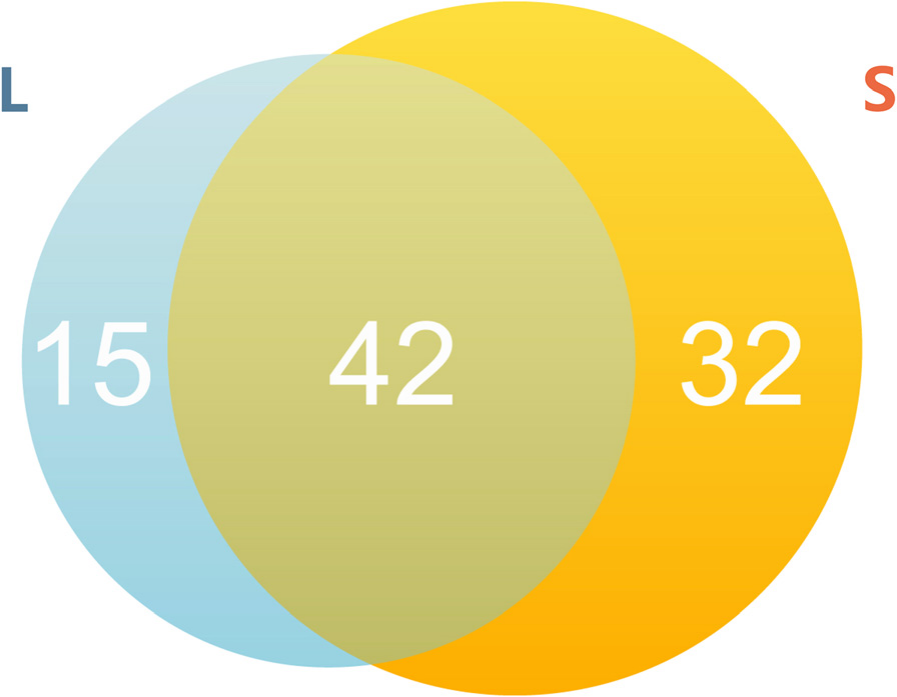
**The peak numbers identified by the program MACS.** 15 peaks are specific for the L phase; 32 peaks are specific for the S phase; 42 peaks are shared by both phases.

**Figure 3:**
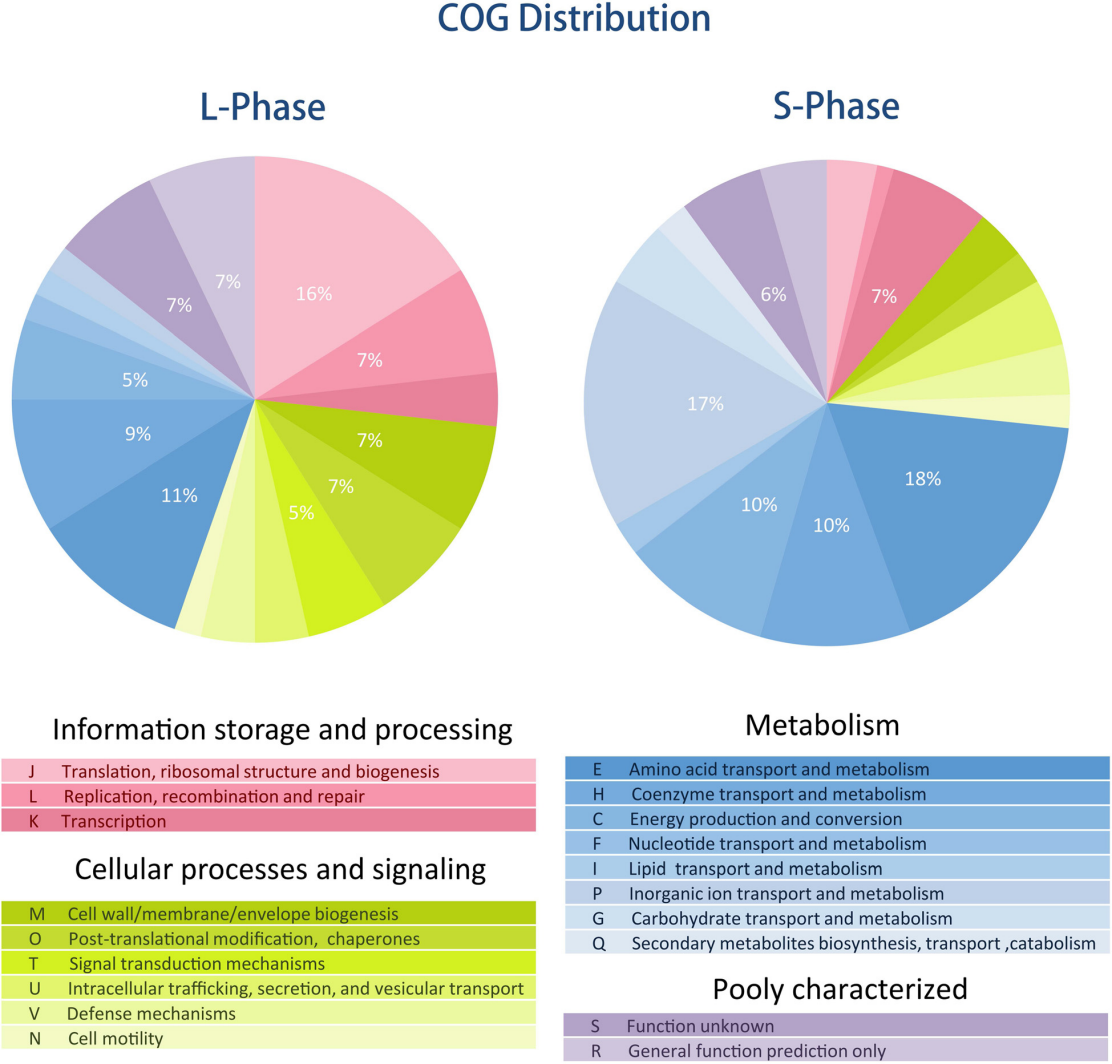
**The proportions of COG functional categories in the highly interacting DNA fragments.** The proportions of COG functional categories for the *E. coli* genes in the highly interacting DNA fragments, measured in the L (left) and S (right) phases. The codes for the COG functions are presented below the pie charts. Percentages given in the pie chart were calculated including categories R and S, and only values ≥5% are shown.

In the genomic interaction profile of the GCC dataset, highly interacting DNA regions can be characterized by the peaks (regions with markedly enriched read densities [15]) in the genomic interaction profile of the GCC dataset. Using the software MACS2 (version 2.0.10) [16], we identified the peaks from the mapping results obtained by Bowtie2 (Figure 1). Totally, there are 89 peaks for L and S phases, with 42 peaks shared, 15 specific for the L phase and 32 specific for the S phase (Figure 2). The gene functions in these peak regions were examined using a cluster of orthologous groups (COG) functional classification scheme (Figure 3, Additional file 3) and it was found that the peak regions for the L phase were enriched with genes of function J (translation, ribosomal structure and biogenesis), E (amino acid transport and metabolism), and H (coenzyme transport and metabolism), and the peak regions for the S phase were enriched with the genes of function E (amino acid transport and metabolism), P (inorganic ion transport and metabolism), and C (energy production and conversion). As a result, these specific gene functions could be interpreted in terms of the physiological states of the two cellular phases. That is, in the L phase, larger amounts of biomass are required for cell growth and proliferation, which require the coordination of the protein translation and biogenesis genes. In the S phase, the transport pathways are highly coordinated as cells struggle to remain alive.

### Spatial features for *E. coli* genome organization

By considering the individual operons in each DNA bin, the interaction frequencies between operons were derived from the interaction information of DNA fragments, and their connections to the operon organization were investigated. The interaction frequencies between operon pairs within a regulon were calculated and compared with those of randomly sampled operon pairs with similar sequence distances (with the same number of operons in between), excluding 0 interaction counts. The interaction frequency of an operon pair belonging to the same regulon was significantly higher than that of a random pair for both the L and S phases (Additional file 1: Figure S2a). Furthermore, the remote operon pairs, whose sequences were separated by at least 100 operons, were also compared with random samples (Additional file 1: Figure S2b). Notably, these remote operons still showed higher interaction frequencies than the randomly sampled operon pairs (with distance >100 operons) from the entire *E. coli* genome. This finding indicated that the DNA interaction-based genome architecture does contribute to the organization of operons into regulons. It also explains the frequent occurrence of the large regulons composed of multiple operons that are sequentially far from each other, thus confirming the suggested functions of 3D chromosome packing on the global organization of operons [6]. We also found a similar phenomenon for genes in biological pathways. The interaction frequency between gene pairs in the same biological pathway was significantly higher than that of gene pairs obtained randomly from the genome for both phases (Additional file 1: Figures S2c, d). This phenomenon was observed not only in the overall gene pairs, but also in the remote gene pairs with sequence separation of at least 100 genes. Taken together, the results suggested that not only operons in a regulon but also genes in a biological pathway were likely to co-localize in the 3D *E. coli* genome.

To examine the spatial features for *E. coli* genome organization quantitatively, the *C* value was defined based on the DNA interaction frequency to measure the organizational compactness of the 3D genome at two levels: the compactness of regulons in terms of the interaction between operon pairs, and the compactness of biological pathways in terms of the interaction between gene pairs. A lower *C* value indicated that the operons/genes are more spread out and less compact in the 3D space globally.

**Figure 4:**
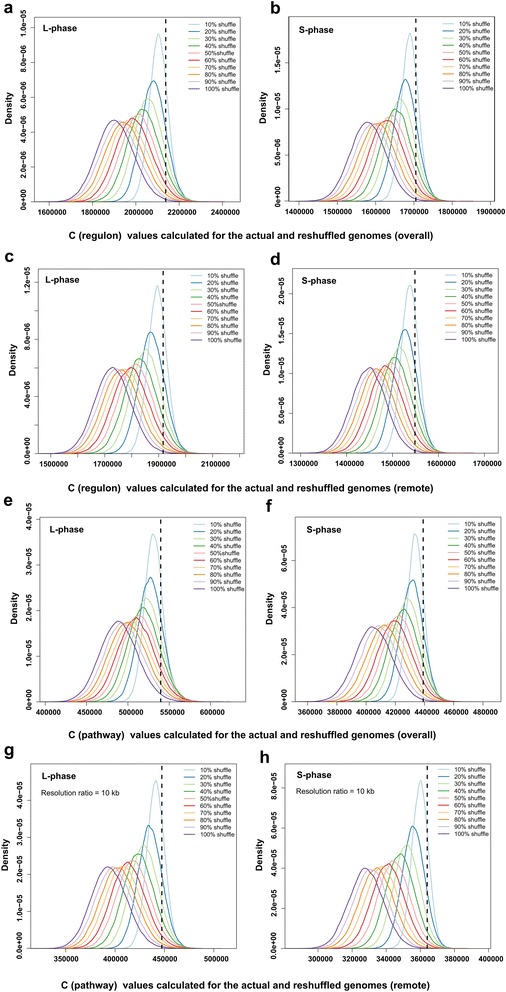
**Distributions of C (compactness) values calculated for the actual and reshuffled genomes.** In each panel, the *x*-axis represents the *C* values of the genome in different conditions (L and S phases); the *y*-axis is the density. Both the “overall” and “remote” operon/gene pairs in the regulons/biological pathways are illustrated respectively, where “remote” means an operon/gene pair whose sequences are separated by at least 100 operons/genes in between on the genome sequence. The black vertical dash line shows the *C* value for the actual arrangement (in the 3D genome) of the overall/remote operons in regulons for the L phase **(a)/(c)** and S phase **(b)/(d)** or the overall/remote genes in the biological pathways for the L phase **(e)/(g)** and S phase **(f)/(h)**. The 10 colored curves in each panel show the distributions of the corresponding *C* values for the randomly reshuffled genomes at different percentages (X% where X = 10, 20… 100, from right to left), respectively. Each colored curve was calculated using 100,000 random permutations of the current arrangement of the considered unit (operon or gene) in the genome.

To determine if the actual genome organization in the 3D space is coordinated compared with random arrangements, the genome was randomly shuffled (totally 1,000,000 times) in different degrees (percentage X = 10, 20, 30… 100), following a procedure similar to that previously reported [5]. We compared the arrangement of operons and genes in the real and randomly reshuffled *E. coli* genomes for both the overall operon/gene pairs and remote ones with sequence separation of at least 100 operons/genes of distance in L and S phases (see Methods). The results showed that the current genomic arrangement of overall operons in genomes had higher *C* values (the vertical dashed lines) than the vast majority of the values in the reshuffled genomes (colored solid lines), in both phases (Figure 4a, b). Moreover, the higher the percentage of randomly reshuffled operons, the larger the decrease in the *C* value of the resulting rearranged genome. The relatively high *C* value for the actual genome arrangement indicated that the actual arrangement of the operons in the regulons in the real genome was more compact than that in the randomly reshuffled genomes. Furthermore, if only the remote operon pairs (with sequence separation of at least 100 operons in between) were considered, this relation persisted (Figure 4c, d). This result indicated that the compactness of the real genome was not just a consequence of the interaction between linearly close operons, but reflects the compactness of *E. coli* genome organization in the 3D space. Meanwhile, the gene arrangement in the biological pathways showed a similar trend. The actual genomic arrangement of the biological pathways had higher *C* values (vertical dashed lines) than the vast majority of those with different extents of reshuffling (colored solid lines), for both the overall and remote gene pairs (Figure 4e, f, g, h).

### Implications for *E. coli* biology

The qualitative and quantitative results both indicated that the previously reported organization principle of *E. coli* genome on the linear sequence [5,6] could be extended to the 3D space. The non-random organization of the linear genome has several effects. For example, neighboring genes on the genome have higher probability of co-expression and their protein products tend to form protein–protein interactions (PPIs) [5,17-21]. Here, we investigated if these effects persist in the 3D space.

**Figure 5:**
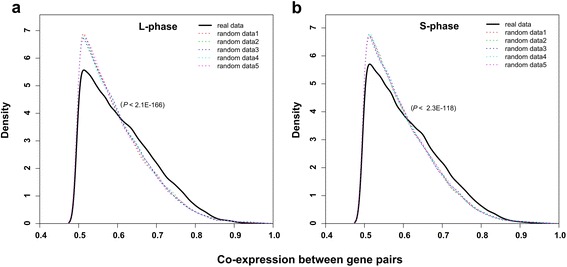
**Co-expression of the remote interacting gene pairs in the L phase (a) and S phase (b).** The top 10% highest interacting remote gene pairs (black solid line), which are located at a distance of at least 100 kb from each other, are compared with the five datasets of randomly sampled remote gene pairs (colored dotted line). *P*-values were calculated using the Wilcoxon rank sum test to compare “real” and “random” data.

We compared the Pearson correlation coefficients (PCCs) of expression levels between the highly interacting gene pairs and randomly sampled gene pairs, using the Wilcoxon rank sum tests. The remote (at least 100 kb far from each other on the genomic sequence) gene pairs with the highest interaction frequency showed a significantly higher co-expression than the five datasets of randomly sampled remote gene pairs, for L (*P* < 2.1e–166) and S (*P* < 2.3e–118) phases (Figure 5). The results suggested the co-expression of genes separated by a long distance on the genome sequence but close to each other in the 3D space.

**Figure 6:**
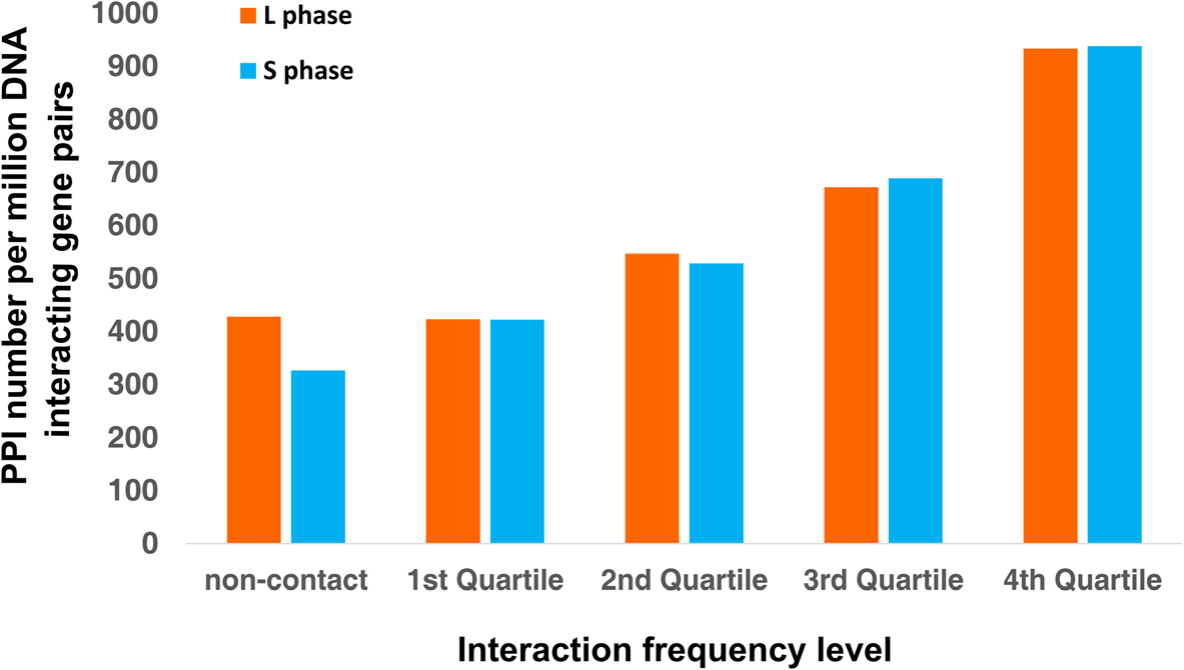
**PPI numbers observed in gene pairs with different DNA-interaction levels.** The *x*-axis shows the first, second, third and fourth quartiles of the DNA-interaction frequency (excluding non-contact) between gene pairs. “Non-contact” represents the gene pairs with no interaction information. The *y-axis* represents the observed PPI number per million DNA-interacting gene pairs. The corresponding *P*-values calculated using the Wilcoxon rank sum test are listed in Additional file 1: Table S3.

To investigate the effect of 3D genome organization on PPI, we compared the PPI occurrence in gene pairs with different levels of DNA interaction frequency. Figure 6 shows that the quartile-based division has higher numbers of PPIs in the highly interacting gene pairs for both phases, with 423.09/422.51 PPI per million DNA-interacting gene pairs in the 1^st^ quartile level and 933.31/937.82 PPI per million DNA-interacting gene pairs in the 4^th^ quartile level, in the L/S phases (the corresponding *P*-values for the comparisons between quartiles are shown in Additional file 1: Table S3). The increasing trend of the column bar height from left to right indicates a positive correlation between the DNA interaction frequency and corresponding PPI frequency of their protein products. This correlation denotes that the proteins encoded by the gene pairs of high DNA interaction in the 3D space have higher probability of forming a PPI. This finding illustrates the connection between the 3D genome organization and bacterial PPI formation.

For bacteria, the processes of transcription, translation, and PPI formation cannot be entirely separated because they lack a nuclear membrane. Thus, the connections observed in this study among the spatial DNA interactions, gene co-expression and protein interactions were partially interpretable in terms of cellular structure. These connections reflect the global genome organization features and the unity of transcription and its downstream processes for *E. coli* in the 3D space, which supports the notion of transcription factory which was modeled for all genomes [22].

## Conclusions

In summary, starting from the GCC data for *E. coli* [13], the present analysis revealed certain spatial features of the *E. coli* genome organization: i) the operons/genes are not randomly distributed in the 3D space, but are constrained by regulons/bio-pathways to maximize spatial compactness; ii) the genes close to each other in the 3D space (even if far from each other on the genome sequence) exhibit trends of co-expression and formation of PPIs. These findings are helpful in elucidating the fundamental biology of bacteria, and support the concept of transcription factory.

## Methods

### Renormalization of the GCC data

The GCC sequencing data for *E. coli* MG1655 at L (exponential sample, WT) and S (serine hydroxamate-treated sample, SHX) growth phases were obtained from the NCBI SRA database. Only the first 70 bp of the whole reads with high quality were mapped onto the *E. coli* reference genome (Accession: NC_000913) using bowtie2 with the default parameters [14]. Unique matches with score > 30 were used for further analysis. The genome was then divided into 32,802 Hhal restriction fragments. The matched RESs in their 500-bp-long flanking sequences were removed as random breaks [23]. The read pairs were further refined by setting a contact distance threshold (>800 bp) between the contact fragment pairs to remove self-ligation and non-ligation [24]. The basic interaction information on the remaining DNA fragments is presented in Additional file 1: Table S1. To differentiate the real contact from background noise, the FDR was controlled [25]. By controlling the FDR at < 0.1, the fragment pairs with at least two contacts are non-random and thus were used for the analysis [26] (see Additional file 2).

Considering the size of the operons and genes, the genome was divided into 10-kb bins, and the interaction frequencies for restriction fragments were assigned to the corresponding bins [12]. *f*_*ij*_ is the interaction frequency between bin *i* and bin *j*. For each bin, the interaction score is defined as the sum of the interaction frequencies in that bin to reflect the interaction potential. We observed a significant, positive correlation between the interaction score and number of Hhal RESs for the GCC dataset (Additional file 1: Figure S1). Therefore, we normalized the interaction frequencies by dividing the number of Hhal RESs for each bin to remove this bias, following the method of a previous report [12]. The interaction matrix after normalization is presented in Additional file 4.

The peaks in the genomic interaction profile were identified using a previously published algorithm [16]. In the algorithm, read distribution along the genome could be modeled by a Poisson distribution [27] in which the parameter λ could capture both the mean and variance of the distribution. Across the genome, we searched for candidate peaks with a significant tag enrichment (Poisson distribution *P*-value based on λ, *P* = 10^-3^ in this work).

### Derivation and handling of pathway and regulon data

The genome sequence and 4,639 annotated genes for *E. coli* were obtained from the NCBI RefSeq. The 319 biological pathways of *E. coli* that involved gene number ≥ 2 were obtained from the EcoCyc database [28]. A total of 2,647 operons and 193 regulons were obtained from the RegulonDB database [29], and the 146 regulons with operon number ≥ 2 were used in our analysis.

For each regulon, the interaction frequencies between operon pairs within it were calculated (excluding 0 interaction counts). The background noise was estimated by randomly sampling operon pairs from the genome, keeping the number of operons between the same as the real interacting operon pairs. Using the Wilcoxon rank sum test, the significance of the real interaction that deviated from the random background was estimated and is shown in Additional file 1: Figure S2. Similarly, the remote operon pairs with a sequence separation of at least 100 operons were also compared with the random background.

To characterize the 3D genome organization quantitatively, we defined an indicator to measure the compactness of the genome in the 3D space, based on the DNA interaction frequency, similar to that in a previous publication [5]:

1 $$ C={\displaystyle \sum_{i=1}^N{c}_i} $$

and

2 $$ {c}_i={\displaystyle \sum_{j=1}^{M_i}{f}_{ij}}, $$

where *f*_*ij*_ is the interaction frequency between a gene/operon pair (*i*, *j*) and is used as a proxy for the 3D distance (the larger the *f*_*ij*_ value, the smaller the distance in the 3D space), *M*_*i*_ is the number of genes/operons in a pathway/regulon, *c*_*i*_ measures the compactness of genome organization in a pathway/regulon, and *C* (the sum of *c*_*i*_) measures the compactness of the whole genome organization in the 3D space.

The genome was then randomly shuffled (totally 1,000,000 times) at different degrees (percentage X = 10, 20, 30, …, 100) following a similar procedure to that previously reported [4] to determine whether the actual genome organization (in terms of interactions between operons/genes in regulons/pathways) in the 3D space is coordinated compared with random arrangements (Figure 4). The comparisons were performed for both the overall operon/gene pairs and the remote ones with sequence separation of at least 100 operons/genes in between.

### Derivation and handling of gene co-expression data

The gene expression data for *E. coli* (E_coli_v4_Build_6; 466 experiments for 4,297 genes) were obtained from the M3D database [30] and the Pearson correlation coefficients (PCCs) were used to measure gene co-expression [31]. The interacting gene pairs that were separated on the genome sequence by at least 100 kb and had the top 10% highest interaction frequencies were used in the co-expression analysis. Two genes were regarded as co-expressed if the PCCs between their expression data were above 0.5 [32,33]. The Wilcoxon rank sum tests were used to compare the distribution of correlation coefficients (of co-expressed genes) between highly interacting gene pairs and the random sampled gene pairs that were at least 100 kb from each other on the genome sequence (Figure 5).

### Derivation and handling of protein–protein interaction data

The protein interaction data for *E. coli* were downloaded from the DIP database (Release 2013.10.31) [34]. For the 12,726 interacting protein pairs obtained from DIP, 8,691 have protein information from the UniProt database (Release 2013_11) [35]. After removing duplicates, 7,345 interacting protein pairs were obtained. The interactions of the proteins whose genes are located on the genome sequence with a distance less than 100 kb were removed. Finally, 6,714 protein interactions were used in the analysis. According to the DNA-interaction frequency, the interacting gene pairs were sorted in ascending order and then classified into four groups (corresponding to four quartiles). With another “non-contact” (interaction frequency = 0) group, five groups of gene pairs were thus used in the comparison of PPI frequency between their protein products. For the 6,714 analyzed protein interactions in *E. coli*, the fractions of these PPI in the five groups of DNA-interacting gene pairs were calculated and plotted in Figure 6 (magnified 1 million times). The differences between the proportions of PPIs in the five groups were compared using Wilcoxon rank sum tests (Additional file 1: Table S3).

## Additional files

Additional file 1: Figure S1. The correlation between DNA interaction counts and the restriction enzyme Hhal site numbers in *E. coli.* The left and right panels correspond to the L-phase and the S-phase. The red line in each panel is the linear fitting. **Figure S2.** Box plot for the comparison of interaction frequencies between real data and random background. The y axis represents the interaction frequency; the four boxes in each sup-graph represent the real interaction in L-phase/S-phase, the random background in L-phase/S-phase respectively. Statistical significance of the difference was calculated by Wilcoxon rank sum test. The “Overall” (a) and sequentially “Remote” (genome sequence separation of at least 100 operons in between) (b) operon pairs in the same regulon; the “Overall” (c) and sequentially “Remote” (genome sequence separation of at least 100 genes in between) (d) gene pairs in the same biological pathway. **Table S1.** The basic information about the DNA fragments and contacts. **Table S2.** False Discovery Rate (FDR) calculations for the genome conformation capture dataset used in this study. **Table S3.** The *P*-value ^a^ of Wilcoxon rank sum test for Figure 6. Additional file 2:
**Supplementary method for the false discovery rate (FDR) calculation.**
Additional file 3:
**Genes in the peak regions of the DNA-interaction profiles for the L and S phases.**
Additional file 4:
**Genome-wide DNA-interaction matrices for L and S phases.** The number in each position (*i*, *j*) in the matrix represents the interaction frequency between bin *i* and bin *j* in the divided genome.
