# Supplementary material for: Spatial features for Escherichia coli genome organization
Source: BMC Genomics. 2015 Feb 5;16(1):37. doi: 10.1186/s12864-015-1258-1 (PMC4326437; doi:10.1186/s12864-015-1258-1)
Supplement: Additional file 1: Figure S1. — The correlation between DNA interaction counts and the restriction enzyme Hhal site numbers in E. coli. The left and right panels correspond to the L-phase and the S-phase. The red line in each panel is the linear fitting. Figure S2. Box plot for the comparison of interaction frequencies between real data and random background. The y axis represents the interaction frequency; the four boxes in each sup-graph represent the real interaction in L-phase/S-phase, the random background in L-phase/S-phase respectively. Statistical significance of the difference was calculated by Wilcoxon rank sum test. The “Overall” (a) and sequentially “Remote” (genome sequence separation of at least 100 operons in between) (b) operon pairs in the same regulon; the “Overall” (c) and sequentially “Remote” (genome sequence separation of at least 100 genes in between) (d) gene pairs in the same biological pathway. Table S1. The basic information about the DNA fragments and contacts. Table S2. False Discovery Rate (FDR) calculations for the genome conformation capture dataset used in this study. Table S3. The P-value a of Wilcoxon rank sum test for Figure 6. [file 12864_2015_1258_MOESM1_ESM.docx]

**
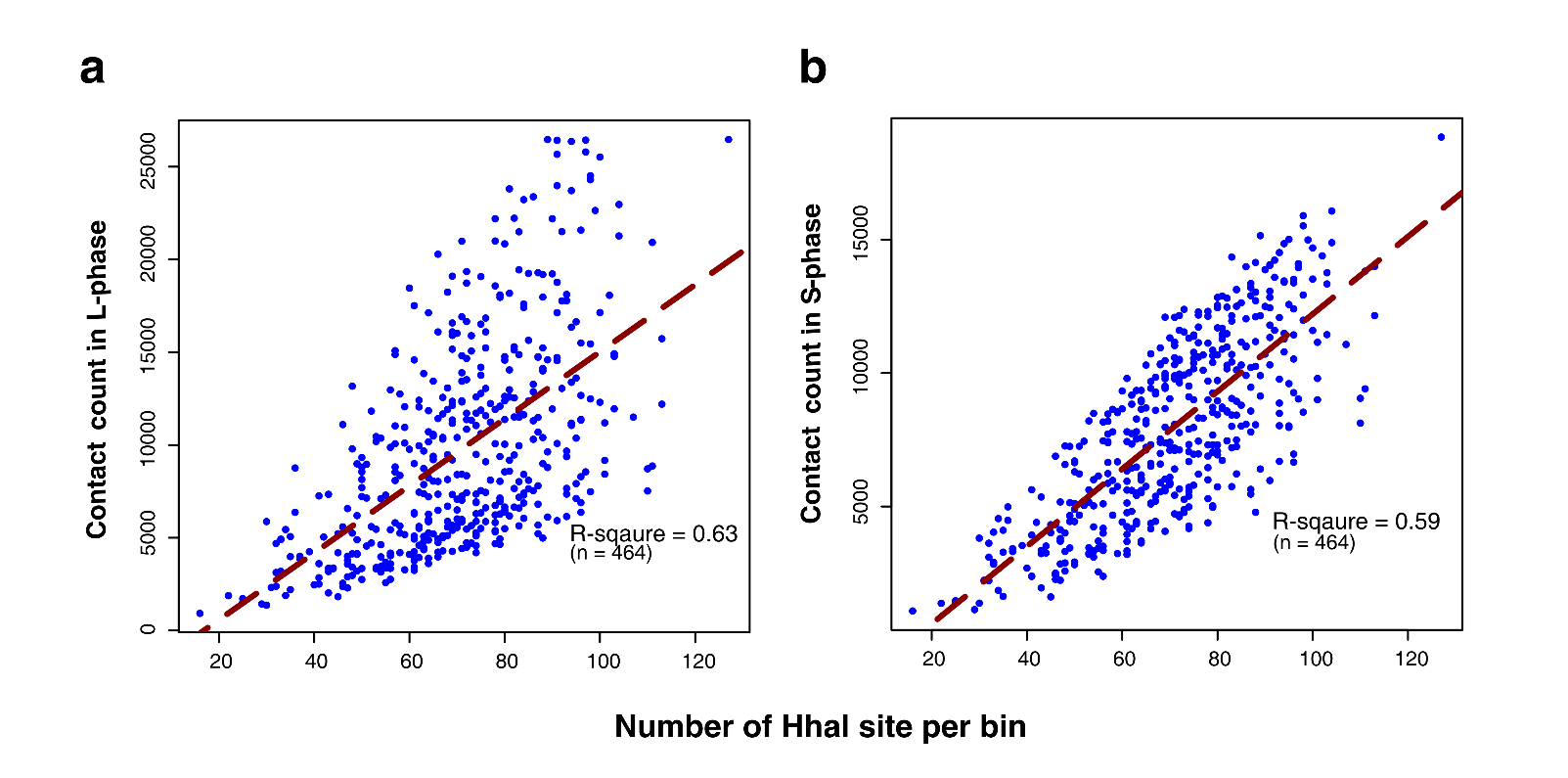
Figure S1. The correlation between DNA interaction counts and the restriction enzyme Hhal site numbers in *E. coli.*** The left and right panels correspond to the L-phase and the S-phase. The red line in each panel is the linear fitting.

**
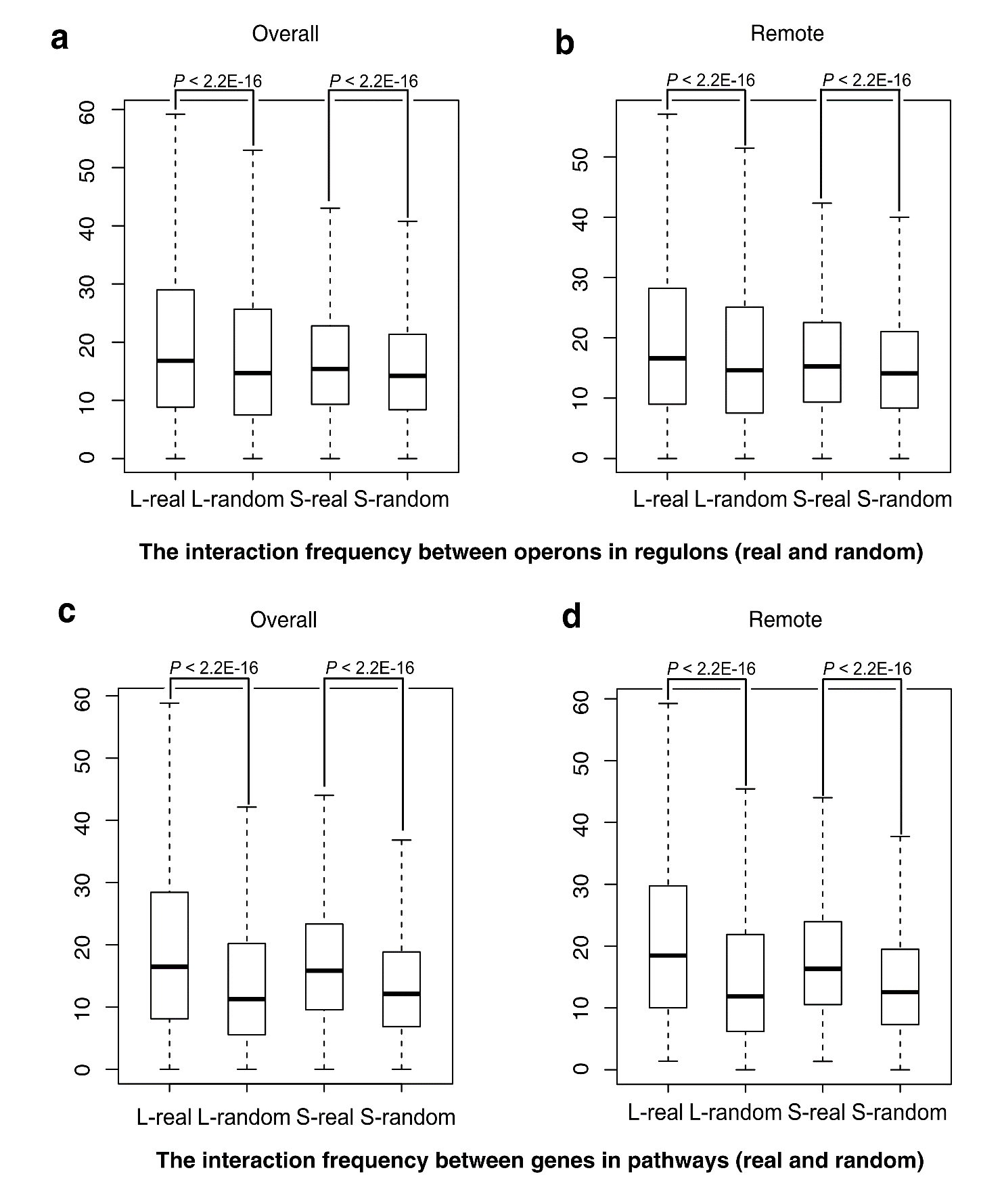
Figure S2. Box plot for the comparison of interaction frequencies between real data and random background.** The y axis represents the interaction frequency; the four boxes in each sup-graph represent the real interaction in L-phase/S-phase, the random background in L-phase/S-phase respectively. Statistical significance of the difference was calculated by Wilcoxon rank sum test. The “Overall” **(a)** and sequentially “Remote” (genome sequence separation of at least 100 operons in between) **(b)** operon pairs in the same regulon; the “Overall” **(c)** and sequentially “Remote” (genome sequence separation of at least 100 genes in between) **(d)** gene pairs in the same biological pathway.

**Supplementary Tables**

**Table S1.** The basic information about the DNA fragments and contacts

| **Phase** | **Raw read  pairs No.** | **Mapped read pairs No.** | **Quality map pairs No. ^a^** | **Random breaks**  **No.** | **Non-ligation &self-ligation No. ^b^** | **Normal ligation No.** | **Effective ligation ^c^ (%) ^d^** |
| --- | --- | --- | --- | --- | --- | --- | --- |
|  |  |  |  |  |  |  |  |
| L | 134,115,349 | 121157985 | 114,714,852 | 3,927,198 | 103,842,610 | 6,945,044 | 2,257,259(1.683%) |
| S | 136,178,353 | 120113842 | 114,338,209 | 3,715,054 | 101,492,726 | 9,130,429 | 1,846,543(1.355%) |

^a^: mapping score > 30.

^b^: the distance of the contacting pair < 800bp.

^c^: the contact counts > 1 for a fragment pair by controlling FDR(see **Table S2**).

^d^: the percentage of the effective contacts in raw reads pairs.

**Table S2.** False Discovery Rate (FDR) calculations for the genome conformation capture dataset used in this study

| **Phase** | **k** | **N** | **p** | **L** | **E** | **n** | **FDR (E/n)** |
| --- | --- | --- | --- | --- | --- | --- | --- |
| L | 0 | 6945044 | 2/34362*34362 | 590373522 | 6904354 | 5559330 | 1.24 |
|  | 1 | 6945044 | 2/34362*34362 | 590373522 | 40531.14 | 871545 | 0.047 |
|  | 2 | 6945044 | 2/34362*34362 | 590373522 | 158.78 | 277230 | 5.73E-04 |
| S | 0 | 9130429 | 2/34320*34320 | 588931200 | 9060017 | 8100965 | 1.12 |
|  | 1 | 9130429 | 2/34320*34320 | 588931200 | 70049.01 | 817079 | 0.086 |
|  | 2 | 9130429 | 2/34320*34320 | 588931200 | 361.53 | 153111 | 2.36E-03 |

k: the required contact count that one fragment pair involved.

N: the total number of observed contact counts.

p:.the probability of each segment to pair to another in theory.

L: the up-limit of the contact number in theory.

n: the number of fragments (X > k).

E: the expected number of false positives (X > k).

**Table S3.** The *P*-value ^a^ of Wilcoxon rank sum test for **Figure 6**

| Species | Phase | Order | Non-contact | First quartile | Second quartile | Third quartile | Forth quartile |
| --- | --- | --- | --- | --- | --- | --- | --- |
| *Escherichia coli K-12* | L | Non-contact | - | 8.70E-01* | 6.40E-04 | 1.20E-10 | 1.00E-30 |
|  |  | First quartile | 8.70E-01^*^ | - | 8.30E-10 | 2.30E-31 | 3.30E-102 |
|  |  | Second quartile | 6.40E-04 | 8.30E-10 | - | 2.60E-08 | 9.80E-55 |
|  |  | Third quartile | 1.20E-10 | 2.30E-31 | 2.60E-08 | - | 5.10E-24 |
|  |  | Forth quartile | 1.00E-30 | 3.30E-102 | 9.80E-55 | 5.10E-24 | - |
|  | S |  |  |  |  |  |  |
|  |  | Non-contact | - | 5.90E-03 | 1.60E-07 | 9.60E-17 | 7.80E-34 |
|  |  | First quartile | 5.90E-03 | - | 7.40E-08 | 9.90E-36 | 1.80E-105 |
|  |  | Second quartile | 1.60E-07 | 7.40E-08 | - | 7.90E-13 | 2.10E-62 |
|  |  | Third quartile | 9.60E-17 | 9.90E-36 | 7.90E-13 | - | 5.90E-22 |
|  |  | Forth quartile | 7.80E-34 | 1.80E-105 | 2.10E-62 | 5.90E-22 | - |

^a^ Asterisk “*” indicates *P*-value > 0.05.
