# Supplementary material for: Spatial features for Escherichia coli genome organization
Source: BMC Genomics. 2015 Feb 5;16(1):37. doi: 10.1186/s12864-015-1258-1 (PMC4326437; doi:10.1186/s12864-015-1258-1)
Supplement: Additional file 2: — Supplementary method for the false discovery rate (FDR) calculation. [file 12864_2015_1258_MOESM2_ESM.docx]

**Supplementary Method**

To differentiate the real contact from background noise, the *false discovery rate* (FDR) was controlled (Rodley *et al.*, 2012). Suppose the occurrence number of interaction between a fragment pair follows the binomial distribution, the contact possibility for each pair is *p* = 2 / *s* * *s* (*s* is the total number of the fragments), and the up-limit of the possible interaction number is *L* = *s* * *s* / 2, then we have

$$FDR(k)=\frac{\left(1-\sum_{i=0}^{k} \left(\begin{matrix} N \\ i \end{matrix}\right)p^{i}(1-p)^{N-i}\right)\times L}{n}$$

where *k* is the lower limit of the required interaction number to control, *N* is the total number of interactions that actually occurred, the numerator $\left(1-\sum_{i=0}^{k} \left(\begin{matrix} N \\ i \end{matrix}\right)p^{i}(1-p)^{N-i}\right)\times L$ is the number of fragment pairs with interaction number greater than *k* expected under random simulation, and the denominator *n* is the number of fragment pairs with interaction number greater than *k* actually occurred. The FDR concerning the different *k* values were calculated based on the aforementioned formula and are listed in **Table S2**. By controlling FDR < 0.1, the minimum *k* value is 1, indicating that the fragment pairs with at least two contacts are non-random and thus used for analysis.
